# Systematic review and meta-analysis of neurofeedback training efficacy and neural mechanisms in the treatment of posttraumatic stress disorder

**DOI:** 10.3389/fnins.2025.1658652

**Published:** 2025-12-03

**Authors:** Daniel E. Berman, Kiriana P. Cowansage, Dawn M. Bellanti, Reshmi Nair, Courtney C. Boyd, Erin H. Beech, Madhavi K. Reddy, Robyn S. Recker, Bradley E. Belsher, Marija S. Kelber

**Affiliations:** 1 Psychological Health Center of Excellence, Defense Health Agency, Falls Church, VA, United States; 2 VA Evidence Synthesis Program Coordinating Center, VA Portland Health Care System, Portland, OR, United States; 3 Research Transition Office, Center for Military Psychiatry and Neuroscience, Walter Reed Army Institute of Research, Silver Spring, MD, United States; 4 TechWerks, LLC, San Antonio, TX, United States; 5 Carl T. Hayden Veterans’ Administration Medical Center, Phoenix VA Health Care System, Phoenix, AZ, United States

**Keywords:** neurofeedback, PTSD, EEG, fMRI, amygdala

## Abstract

**Introduction:**

Neurofeedback in the treatment of psychological disorders has gained increasingly widespread interest in recent years. As the use of neurofeedback training expands, it is important to elucidate its treatment efficacy, especially for prevalent and debilitating psychopathologies such as posttraumatic stress disorder (PTSD). Likewise, furthering our understanding of the underlying neural mechanisms by which neurofeedback acts is also necessary.

**Methods:**

Here, we present the results of a PROSPERO registered (protocol number: CRD42020184659) meta-analysis of randomized controlled trials (RCTs) of neurofeedback training for treatment of PTSD in adults. We evaluate the efficacy of different neurofeedback modalities used to treat PTSD, including functional magnetic resonance imaging neurofeedback (fMRI-NF) and electroencephalogram neurofeedback (EEG-NF). We also differentiate active control (sham or yoked sham) studies from passive control (waitlist, treatment as usual, no treatment, and any non-neurofeedback based intervention) studies.

**Results:**

Our results show that EEG-NF has a moderate to large effect in reducing PTSD symptoms pre- to post-treatment compared to passive controls (k = 5). Two fMRI-NF RCTs, both using sham controls, showed no improvement in PTSD symptoms, pre- to post-treatment. However, our confidence in these findings is very low to low due to concerns regarding risk of bias, imprecision, and conflicts of interest. Neurofeedback in passive control studies outperformed neurofeedback in active control studies relative to their respective control treatment arms. We also synthesized the neural results from these studies and found that between-group neural effects were generally inconclusive.

**Discussion:**

These findings highlight the need for improved controls in studies examining neurofeedback for PTSD to reliably determine whether neurofeedback training, or other factors, are the basis for improvements in PTSD symptoms. We elaborate on some of the underlying neural mechanisms by which neurofeedback training shows potential in improving PTSD symptoms to guide future developments and provide recommendations for future neurofeedback interventions in treating PTSD.

## 1 Introduction

Posttraumatic stress disorder (PTSD) is a mental health condition that may occur in response to a traumatic event. Individuals with PTSD experience intrusive distressing memories or dreams of the traumatic event, avoidance of stimuli associated with the traumatic event, negative alterations in mood and cognition, and alterations in arousal (e.g., irritable or self-destructive behavior or hypervigilance) (American Psychiatric Association, 2022). Among individuals with PTSD, these symptoms cause significant distress or impairments in social, occupational, or other areas of functioning. Lifetime prevalence of PTSD in the US general population is 4% in men and 8% in women (Goldstein et al., 2016). Due to occupational exposures such as combat, PTSD is more prevalent among service members and veterans than the general population. Lifetime prevalence of PTSD in veterans is 6.2% among men and 13.2% in women (Lehavot et al., 2018). A representative survey of US service members found that 10.4% of active component and 9.3% of reserve component reported probable PTSD in the past 30 days (Meadows et al., 2021).

Many interventions have been developed to treat PTSD. Trauma-focused psychotherapies, such as cognitive processing therapy, prolonged exposure, and eye movement desensitization and reprocessing have the strongest evidence of effectiveness (Va/DoD Clinical Practice Guideline, 2023). However, a substantial number of patients drop out prematurely or do not respond to PTSD psychotherapy (Swift and Greenberg, 2014; Steenkamp et al., 2015; Kehle-Forbes et al., 2016; Resick et al., 2017; Edwards-Stewart et al., 2021). Precise numbers vary depending on how response and dropout are operationalized, but reviews of PTSD psychotherapies have found nonresponse rates of 39% overall (Semmlinger et al., 2024) and 50% to 72% in military/veteran populations (Steenkamp et al., 2015) with dropout rates of approximately 16% overall (Lewis et al., 2020) and 36% in veteran populations (Goetter et al., 2015). These limitations of current psychotherapy treatments for PTSD have prompted researchers to evaluate alternative treatments. Among these alternative treatments, one promising avenue for treating PTSD is neurofeedback training (Bisson et al., 2019).

Neurofeedback training for the treatment of PTSD, and mental health disorders more broadly, is based on a few key principles. First and foremost is that neurophysiology and mental health disorders are strongly linked (Murphy and Bassett, 2017). In PTSD, this may involve increased amygdala activation in response to threat (Alexandra Kredlow et al., 2022), dysregulation of various connectivity networks (Menon, 2011; Malejko et al., 2017), or other neural correlates that are described more thoroughly below. Also, successful neurofeedback training requires that volitional control or implicitly learned modulation of neural processes is possible in response to feedback. This is in line with biofeedback principles more generally, where the learned feedback increases the awareness of an underlying biological process, enabling conscious engagement with that process. Additionally, certain neurophysiological functions are malleable with training and operant conditioning; the act of observing neural signals while cognitive processes occur allows for tuning of that processing. Finally, this learned neuromodulatory capability is transferrable to real-world settings (Krause et al., 2024).

Neurofeedback training methodologies vary widely, but there are several design components that can be found across all neurofeedback paradigms. First, a neural measure of cognitive processes related to the disorder or condition of interest must be chosen. Depending on the modality, this may be a particular bandwidth of electroencephalogram (EEG) waveforms or blood-oxygenation-level-dependent (BOLD) response of a localized brain region (Weiskopf, 2012; Marzbani et al., 2016). The signal from this neural measure is processed in real-time (Cox et al., 1995) during the training protocol and provided to the participant typically in the form of visual stimuli, auditory stimuli, or both (Turk-Browne, 2021). Participants are instructed to modulate this visual or auditory signal in a particular direction chosen by the experimenters or clinicians. Instructions can include explicit strategies for signal modulation, such as “try to relax and recall happy memories” (Zotev et al., 2018) or it can be left up to the participant to devise their own strategy. Neurofeedback for PTSD treatment may also incorporate listening to personalized traumatic scripts while the participant modulates the feedback signal (Fruchtman-Steinbok et al., 2021; Zhao et al., 2023).

The underlying neural correlates for a disorder as complex as PTSD involve numerous, widely distributed cortical and subcortical areas. A prominent feature of PTSD is that it involves increased amygdala activity which is often accompanied by decreased activation of prefrontal cortex (PFC) (Gilboa et al., 2004; Shin et al., 2005; Etkin and Wager, 2007; Mahan and Ressler, 2012; Brown et al., 2014; Dahlgren et al., 2018; Alexandra Kredlow et al., 2022). Emotional regulation and arousal are known to involve amygdala-ventromedial PFC (vmPFC) pathways (Etkin and Wager, 2007; Koenigs and Grafman, 2009; Stevens et al., 2013; Pennington et al., 2017) but other delineations of PFC have also been implicated in PTSD including dorsolateral PFC (dlPFC) (Lyoo et al., 2011) and dorsomedial PFC (dmPFC) (Weisholtz et al., 2021). The evidence suppleting the involvement of the amygdala and PFC in emotional regulation has provided a strong theoretical basis for alleviating symptoms of PTSD using neurofeedback training targeting these regions (Zotev et al., 2011; Gerge, 2020). Likewise, individuals with PTSD have been shown to have reduced or dysregulated alpha rhythms (Jokic-Begic and Begic, 2003) which are associated with hypervigilance symptoms of PTSD (Clancy et al., 2017). Also, attenuated resting-state alpha posterior-frontal connectivity, which may contribute to sensory disinhibition, has been associated with intrusive reliving of traumatic experiences in combat veterans (Clancy et al., 2020a). As such, randomized clinical trials (RCTs) involving neurofeedback training for the treatment of PTSD have generally focused on these regions and bandwidths.

As shown in network models of psychopathology, intrinsic connectivity networks (ICNs) are also implicated in PTSD (Buckner et al., 2008; Menon, 2011). Briefly, the default mode network (DMN), typically most active during rest and less involved in goal-oriented tasks, has been shown to have weaker connections and hypoactivity in patients with PTSD (Koch et al., 2016; Akiki et al., 2017; Malejko et al., 2017). Likewise, hypoactivity within the central executive network (CEN), which regulates decision-making, inhibition, and other high-level cognitive functions (Lanius et al., 2015; Akiki et al., 2017) has been shown to be associated with PTSD (Lanius et al., 2015; Holmes et al., 2018). The salience network (SN), which modulates attention in response to relevant stimuli and regulates network switching (Szeszko and Yehuda, 2019; Schimmelpfennig et al., 2023) has been shown to exhibit increased connectivity between several main hubs of this network, including the amygdala, dorsal anterior cingulate (dACC), and anterior insula (Rabinak et al., 2011; Shin et al., 2011; Brown et al., 2014). Because the SN is fundamental in processing threats, hyperactive connections between these areas can result in the hyperarousal symptoms seen in PTSD, manifested as an ongoing state of vigilance or a reduced threshold for perceived threats (Akiki et al., 2017; Szeszko and Yehuda, 2019).

A principled approach to treating PTSD through neurofeedback should address these neural correlates. Most widely used is electroencephalogram neurofeedback (EEG-NF). Here, neuronal activity is recorded via electrodes placed on the scalps of participants, who are then asked to modulate this activity in response to stimuli and external feedback. EEG-NF has shown promise in treating alcohol use disorder (Dousset et al., 2020), major depressive disorder (Dobbins et al., 2023), eating disorders (Bartholdy et al., 2013), attention deficit hyperactivity disorder (Arns et al., 2009, 2014), and seizure disorders (Sterman, 2000), among other psychopathologies. Several EEG-NF protocols aimed at alleviating symptoms of PTSD have been examined. These procedures are based on established correlations between distinct functional and neurocognitive states and specific frequency components of synchronized neuronal activity. Alpha/theta training is intended to encourage a state of relaxation by promoting alpha (8–12 Hz) and theta (4–7 Hz) band activity (Peniston and Kulkosky, 1991). More precisely, the alpha/theta protocol promotes the ratio of theta to alpha activity to the point where theta activity becomes more dominant than alpha (Egner et al., 2002). Thus, the theoretical basis for this treatment is that by promoting lower frequency brain states when exposed to traumatizing stimuli, previously anxiety- or fear-inducing neural circuitry can be reconditioned (Kluetsch et al., 2014). Alpha down training protocols follow similar logic as alpha/theta training, except the emphasis is solely on the attenuation of alpha activity (Ros et al., 2013; Nicholson et al., 2020). Finally, infra-low frequency neurofeedback (ILF-NF) training, involves the modulation of slow cortical potentials below 0.1 Hz as a means to implicitly control overall nervous system excitability and improve arousal regulation (Othmer et al., 2013; Bazzana et al., 2022).

Neurofeedback using functional magnetic resonance imaging (fMRI) has also gained popularity in recent years. fMRI is used to measure the BOLD response throughout the brain, and fMRI neurofeedback (fMRI-NF) is aimed at modulating the BOLD signal, typically in a target region functionally linked to the underlying symptomology of the condition being treated (Weiskopf, 2012). Greater precision of neural targets is the main benefit of fMRI-NF over EEG-NF. The BOLD signal from a target region of interest (ROI) is processed in real-time and is used as the feedback signal that the participant attempts to modulate. The target ROI most relevant for this review is the amygdala, but fMRI-NF for PTSD has also been explored using the lateral PFC (Zweerings et al., 2020) and posterior cingulate cortex (PCC) (Lieberman et al., 2023).

To mitigate the downsides of fMRI, particularly cost and accessibility concerns, while leveraging the improved spatial resolution that fMRI offers, more elaborate techniques have been developed in recent years that combine fMRI data with EEG. Most relevant for this review is the fMRI-inspired EEG technique known as amygdala-electrical fingerprint (amyg-EFP) (Keynan et al., 2016; Keynan et al., 2019). Briefly, amyg-EFP allows EEG signals to act as a proxy for measuring activity of the amygdala. Keynan et al. (2016) show that despite the poor spatial resolution of EEG, it is possible to model the amygdala’s BOLD response as measured by fMRI, using a single electrode (Pz) and minimal assumptions. Despite its nomenclature, amyg-EFP does not strictly act as a proxy for amygdala activity alone, rather it is a proxy of a greater network in which the amygdala participates, including subgenual ACC and insula (Keynan et al., 2016). These regions implicated in amyg-EFP overlap with the SN and modulation of the entire network does have implications for PTSD symptom alleviation (Lanius et al., 2015; Akiki et al., 2017).

A recent meta-analysis that pooled neurofeedback types (EEG-NF and fMRI-NF) across existing RCTs found that neurofeedback reduced PTSD symptoms from pre- to post-treatment on both the Clinician-Administered PTSD Scale (CAPS) and PTSD Checklist (PCL) with a high quality of evidence rating (Voigt et al., 2024). The authors’ overall favorable impression of the neurofeedback treatment for PTSD should be taken cautiously given their potential conflict of interest. Furthermore, to gauge whether the effects of neurofeedback training are the result of neurofeedback or are rooted in placebo, findings must be interpreted in with study design and the type of control used (Thibault and Raz, 2017; Sorger et al., 2019). For example, active controls like sham and yoked sham comparators provide a true placebo condition not afforded by passive controls such as waitlist and treatment as usual comparators (Sorger et al., 2019). Given all of these factors, a thorough and conflict free analysis of the extant literature is warranted. There is also a gap in the literature regarding the neural outcomes found in neurofeedback training for PTSD RCTs that needs to be filled. We provide a brief meta-review of some relevant literature that our work builds upon in Table 1.

**TABLE 1:** Meta-review of previous systematic reviews on neurofeedback for posttraumatic stress disorder.

References	Included study types	Modalities included	Meta-analysis component	Findings/conclusions	Considerations
Askovic et al., 2023	NRSIs and RCTs	EEG-NF	Yes	Large effect size in favor of EEG-NF; very low to moderate certainty of evidence	EEG-NF only and inclusion of NRSIs
Chiba et al., 2019	NRSIs, open trials and RCTs	EEG-NF and fMRI-NF	Yes	DecNef showed substantial PTSD symptom amelioration in preliminary trial; efficacy of neurofeedback to date is limited and could benefit from advanced techniques	Review and meta-analysis were secondary aims; significant conflict of interest
Hong and Park, 2022	RCTs	EEG-NF and fMRI-NF	Yes	EEG-NF shows moderate effect in PTSD symptom reduction; lower doses of neurofeedback tended to be better than higher dosage, but dose-response findings were inconclusive	Failure to link studies using same sample; missed several recent RCTs
Matsuyanagi, 2025	NRSIs and RCTs	EEG-NF	Yes	EEG-NF is effective in reducing PTSD symptoms; publication year is significant moderator suggesting that as study designs improve (e.g., more sham-controls) effects decrease	Failure to link studies using same sample; EEG-NF only
Steingrimsson et al., 2020	RCTs	EEG-NF	Yes	Findings of the four small studies suggest that treatment with EEG-NF may improve PTSD symptoms in adult patients with PTSD	Inclusion of only four studies, latest from 2017
Panisch and Hai, 2020	Exploratory pilots, NRSIs, and RCTs	EEG-NF and fMRI-NF	No	Ten studies showed at least one outcome measure of PTSD symptom improvement	Inclusion of six studies without controls
Pindi et al., 2022	NRSIs and RCTs; all MH disorders	fMRI-NF	No (for PTSD)	fMRI-NF for MH disorders can be effective but high heterogeneity if effect sizes and methodology between studies temper findings	Narrative results for PTSD studies only
Voigt et al., 2024	RCTs	EEG-NF and fMRI-NF	Yes	Large effect size in favor of neurofeedback; high certainty and quality of evidence and overall low risk of bias	Significant conflict of interest; concerns with effect size calculations and other methodological aspects; non-adult samples; no synthesis of neural results

EEG-NF, electroencephalogram neurofeedback; fMRI-NF, functional magnetic resonance imaging neurofeedback; MH, mental health; NRSI, non-randomized studies of intervention; RCT, randomized controlled study; PTSD, posttraumatic stress disorder.

Clinical practice guideline for the management of PTSD by the Department of Defense and Veterans Affairs states that there is insufficient evidence to recommend for or against neurofeedback for the treatment of PTSD (Va/DoD Clinical Practice Guideline, 2023). A thorough review on neurofeedback research has the potential to identify promising neurofeedback modalities and neural targets and identify gaps for future research. Recent reviews have not explored how neurofeedback modality impacts the efficacy of neurofeedback for PTSD treatment, nor have they examined the neural outcomes of the extant literature. Thus, the goal of this systematic review and meta-analysis was to provide such a thorough overview by examining the efficacy of neurofeedback treatment for adults with PTSD in RCTs by addressing two key questions (KQs). KQ1: What is the efficacy of neurofeedback training on PTSD outcomes among adults with PTSD relative to active controls (sham or yoked sham comparators) and passive controls (any control without sham neurofeedback comparators such as waitlist, treatment as usual, no treatment, or other treatments) using different neurofeedback modalities (EEG-NF and fMRI-NF)? KQ2: What are the effects of neurofeedback training on relevant neural outcomes (e.g., network connectivity changes, modulation of ROIs, EEG amplitude changes)?

## 2 Materials and methods

### 2.1 Protocol and search strategy

This systematic review (PROSPERO Protocol number: CRD42020184659) adhered to the Preferred Reporting Items for Systematic Reviews and Meta-Analyses (PRISMA) guidelines (Page et al., 2021). Since publishing the protocol, several systematic reviews have been published on the topic (Askovic et al., 2023; Voigt et al., 2024). Consequently, we have made the following changes to the protocol in order to appropriately close evidence synthesis gaps in this literature and answer our key questions: (1) focused on efficacy of neurofeedback by excluding non-randomized studies of intervention; (2) examined differential effects of EEG-NF and fMRI-NF; and (3) emphasized synthesizing neural results rather than secondary outcomes (e.g., quality of life, adverse events, satisfaction).

A library specialist searched a combination of controlled vocabulary terms and keywords related to the concepts of neurofeedback and PTSD across four databases (PubMed, PsycInfo, Embase, PTSDpubs) from inception to February 2025. Variations of the complete PubMed Search (see Supplementary Appendix A) were conducted across PsycInfo, Embase, and PTSDpubs. The team also consulted with subject matter experts to identify additional relevant publications not captured by the database searches.

### 2.2 Eligibility criteria

The team included articles that met all of the following criteria: peer-reviewed research published in English; randomized controlled trial (RCT) or secondary analysis of RCT design as long as comparisons were made between the randomized groups; adult population (minimum 80% of sample aged 18 or older) with a primary, clinician administered, diagnosis of PTSD according to DSM-IV, DSM-5, ICD-10, or ICD-11 criteria; intervention consisting of neurofeedback delivered via EEG and/or fMRI imaging with therapeutic intent; at least one comparator group (i.e., sham neurofeedback, treatment as usual, waitlist, no treatment, other treatments) diagnosed with PTSD; and at least one of the following outcome categories reporting between-group intervention effects: (i) PTSD symptoms; (ii) PTSD remission rates; or (iii) neurophysiological results.

### 2.3 Screening and data extraction

Using the pre-defined eligibility criteria, the review team conducted dual, independent screening of titles and abstracts, resolving disagreements through discussion and consensus. Full-text articles were obtained for records marked for inclusion at the title/abstract stage and were then dually screened using the same process. A single reviewer used a customized data extraction form developed by the study team to extract the study characteristics, description of the neurofeedback intervention, and findings for each study and a second reviewer independently verified results. Screening and data extraction was performed using Covidence, a web-based collaborative platform that helps to streamline systematic reviews. One study initially met inclusion criteria but was subsequently excluded due to its cross-over design and the absence of any post-treatment outcome measures prior to exposure to the second treatment arm (Zweerings et al., 2020).

### 2.4 Study quality and certainty of evidence assessment

The review team judged the methodological quality of each study (i.e., sequence generation, allocation concealment, blinding of participants and personnel, blinding of outcome assessors, completeness of outcome data, selective outcome reporting, and other bias) using the Cochrane Risk of Bias Tool (Higgins et al., 2024). Each study was assessed by two raters, and discrepancies within each pair of raters were resolved through discussion. Additionally, Grading of Recommendations Assessment, Development and Evaluation (GRADE) was conducted. This is a systematic approach used to assess and summarize the evidence of findings and provide a confidence rating (very low to high, see descriptions below Table 3) in the findings. Because all studies included here are RCTs, each finding starts with a rating denoting a high certainty of evidence and is downgraded when there are concerns such as considerable risk of bias (Guyatt et al., 2011e), inconsistency (Guyatt et al., 2011b), or imprecision (Guyatt et al., 2011a), and upgraded when other factors provide additional support for the outcome [e.g., a dose-response effect (Guyatt et al., 2011d)]. This was done for active control fMRI-NF (k = 2), active control EEG-NF (k = 1), and passive control EEG-NF (k = 5) studies. We also assessed each included study for adherence to the Consensus on the Reporting and Experimental Design of clinical and cognitive-behavioral neurofeedback studies (CRED-nf) best practices checklist (Ros et al., 2020). CRED-nf guidelines were developed to improve reporting practices and methodology in neurofeedback research. Each study was assessed by two raters, and discrepancies within each pair of raters were resolved through discussion. Results of this assessment can be found in the Supplementary material.

### 2.5 Statistical analysis

We considered studies that used the same participant sample to be the key unit of interest and linked published reports of the same study together, in keeping with the Cochrane Collaboration’s standards for conducting systematic reviews (Li et al., 2019). As such, we linked three studies (Nicholson et al., 2020, 2023; Shaw et al., 2023) and another three publications together (Zotev et al., 2018; Misaki et al., 2018b,^2019^) as two discrete units of analysis (see Table 2).

**TABLE 2:** Characteristics of included randomized controlled trials of neurofeedback training for PTSD.

References (country)	Population description; mean age (SD)^1^; % male	*N* (enrolled/ completed)	Neurofeedback protocol				Control groups	PTSD measure Results: SMD (95% CI)
			NF modality	NF description	NF target (ROI or electrodes)	NF dosage (*N* sessions, total minutes)		
Fine et al., 2023 (Israel)	Women with CSA and diagnosis of PTSD; 37.37 (11.45); 0%	T: 40/35 C: 15/15	EEG	fMRI-informed EEG model of amygdala activity (Amyg-EFP-NF)	Pz (proxy for amygdala)	10 sessions, ∼150 min	Passive: TAU (psychotherapy)	CAPS-5 −0.502 (−1.120, 0.116)
Fruchtman-Steinbok et al., 2021 (Israel)	Civilians with diagnosis of PTSD; 40.25 (21.96), 41.7%	T1: 20/13 T2: 19/14^2^ C: 20/13	EEG	fMRI-informed EEG model of amygdala activity (Amyg-EFP-NF) T1: Received individualized trauma narrative T2: Received neutral auditory stimulus (repetitive jazz music)	Pz (proxy for amygdala)	15 sessions, 225 min	Passive: TAU	CAPS-5 −0.862 (−1.559, −0.164)
Leem et al., 2021 (South Korea)	Civilians with diagnosis of PTSD; 44.40 (13.61); 10%	T: 11/10 C: 11/9	EEG	Alpha/theta EEG	Pz	16 sessions, 480 min	Passive: WLC	PCL-5-K −1.529 (−2.552, −0.505)
Nicholson et al., 2020 (Canada)^3^, Nicholson et al., 2023 (Canada)^4^, Shaw et al., 2023 (Canada)^4^	Civilian, military, first responders with primary diagnosis of PTSD; 40.28 (12.21); 33.3%	T: 18/18 C1: 18/18 C2: 36/NA	EEG	Alpha-rhythm EEG-NF	Pz	20 sessions^5^, 380 min	Active: Yoked sham NF Passive: WLC	Normalized to CAPS-5^6^ *CPSTABLEENTER*−0.430 (−1.091, 0.231)
Noohi et al., 2017 (Iran)	Individuals with war-related trauma and diagnosis of PTSD; ND; 100%	T: 15/15 C: 15/15	EEG	Alpha/theta EEG	Pz	25 sessions, ∼750–1,000 min	Passive: No treatment	IES-R, Farsi version −2.613 (−3.588, −1.639)
van der Kolk et al., 2016 (US)	Civilians diagnosed with PTSD and completed 6 months of trauma-focused psychotherapy; 46.04 (12.89); 7.4%	T: 28/22 C: 24/22	EEG	Inhibit low frequency (theta and delta, 2–6 Hz) and high frequency (high beta, 22–36 Hz) activity while increasing mid-range (alpha, 10–13 Hz) activity	T4	24 sessions, ∼720 min	Passive: WLC	CAPS-IV −1.440 (−2.104, −0.777)
Winkeler et al., 2022 (Germany)	Civilians with comorbid eating disorders and PTSD; 27.11 (5.28); ND	T: 18/17 C: 18/11	EEG	ILF-EEG	T4-P4, T3-T4, and T4-Fp2	12 sessions, 360 min	Passive: Media-supported relaxation	IES-R^7^
Zotev et al., 2018 (US)^3^ Misaki et al., 2018b (US)^4^, Misaki et al., 2019 (US)^4^	Veterans with combat-related PTSD as primary diagnosis; 31(6); 100%	T: 20/15 C: 11/8	fMRI	Upregulation of left amygdala BOLD signal during positive emotion induction task (e.g., happy autobiographical memories)	Left amygdala	3 sessions, 32 min	Active: Sham NF (LHIPS)	CAPS-IV −0.303 (−1.166, 0.559)
Zhao et al., 2023 (US)	Civilians with diagnosis of chronic PTSD; 40.2 (14.27); 21.43%	T: 15/14 C: 12/11	fMRI	Downregulation of amygdala BOLD signal during personalized trauma script	30 most active voxels in amygdala during trauma script	18 sessions, 90 min	Active: Yoked sham NF	CAPS-5 0.169 (−0.622, 0.960)

Amyg-EFP-NF, amygdala electrical fingerprint neurofeedback; C, control group; C1, first control group; C2, second control group; CI, confidence interval; CSA, childhood sexual abuse; DSM-IV-TR, Diagnostic and Statistical Manual of Mental Disorders; EEG, electroencephalogram; ETI-TS, Essener Trauma-Inventar-Traumasymptomatik; fMRI, functional magnetic resonance imaging; IES-R, Impact of Event Scale-Revised; ILF, infra-low frequency; LHIPS, left horizontal segment of intraparietal sulcus; ND, not described; NF, neurofeedback; PCL-5-K, PTSD Checklist for DSM-5- Korean Version; PTSD, posttraumatic stress disorder; Pz, midline parietal cortex; ROI, region of interest; SMD, standardized mean difference; T, treatment group; TAU, treatment as usual; WLC, waitlist control. ^1^Mean age (SD) of primary treatment group, ^2^treatment groups combined for meta-analysis, ^3^primary study, ^4^secondary studies using same sample as primary,^ 5^initial session did not have NF administered, ^6^scores were normalized to the CAPS-5 scale to compare CAPS-5 and earlier CAPS-IV assessments, ^7^unable to calculate SMD based on available data as only the avoidance subscale of the IES-R is provided.

We conducted univariate meta-analyses of standardized pre-post treatment mean differences in PTSD outcome measures. Treatment effects were estimated by computing standardized mean differences (i.e., Cohen’s *d*) from pre-post change score (see Supplementary Appendix B). Many of the included studies reported follow-up measures at varying timepoints after completion of neurofeedback training. As these timepoints were temporally disconnected from the intervention by as much as six months, our analyses focus only on pre-treatment and immediate post-treatment outcomes. Additionally, because neural outcomes were rarely measured during follow-up periods, there was an inability to link PTSD symptom outcomes with neural correlates at these extended time periods. For any instances where these measures can be linked, we narratively describe this.

To address KQ1, analyses were conducted separately for each combination of control comparison (active control or passive control) and modality (EEG-NF or fMRI-NF) that was possible. These main analyses include the following groupings: (1) five studies comparing EEG-NF versus passive control and (2) two studies comparing fMRI-NF and active control. No fMRI-NF studies used a passive control.

One study that compared EEG-NF to active control (Nicholson et al., 2020, 2023; Shaw et al., 2023) was not included in the main meta-analyses to minimize heterogeneity of the control conditions between studies, in line with the basis of KQ1. We compute the effect size for this study and synthesized the results narratively. We also performed two additional analyses, one where this study was included with the five passive control EEG-NF studies, (EEG-NF versus any control), and one where this study was grouped with the two other studies using an active control (any neurofeedback modality versus active control). These additional analyses help to further separate the influence of neurofeedback modality and the type of control on outcomes.

One study (Fruchtman-Steinbok et al., 2021) reported two treatment outcomes (Trauma and Neutral) compared to the same control group. To handle dependence from multiple group comparison, we computed a combined mean and standard deviation (SD), across both Trauma and Neutral groups and used this mean and SD with the mean and SD of the control group in the final meta-analysis (Scammacca et al., 2014). To account for correlation between pre-post repeated measures, we conducted sensitivity analyses across three sets of correlation strengths (high = 0.7, medium = 0.5, low = 0.3). In each meta-analysis, we estimated summary effect sizes using a random effects model and evaluated statistical heterogeneity using τ^2^ and *I*^2^ estimates. We conducted all analyses using Comprehensive Meta-Analysis Software version 4 (Borenstein, 2022).

To examine if differing treatment duration (dose response) might be a potential effect modifier, we conducted a meta-regression within the EEG-NF versus passive control studies (Deeks et al., 2019). This was the only grouping with a sufficient number of studies to conduct this analysis while maintaining adequate homogeneity in control conditions. For each of the five EEG-NF studies using a passive control, we created a dichotomous variable indicating shorter or longer treatment duration using a median split. The study with treatment duration that equaled the median (Leem et al., 2021) was closer in duration to the longer treatment studies and was thus included in the longer treatment group. This dichotomous variable was entered into the meta-regression model with shorter duration as the reference group. The regression coefficient provided an estimate of how the intervention effect in the longer duration group differed from the shorter duration group. Findings on neural outcomes were narratively synthesized.

## 3 Results

**FIGURE 1:**
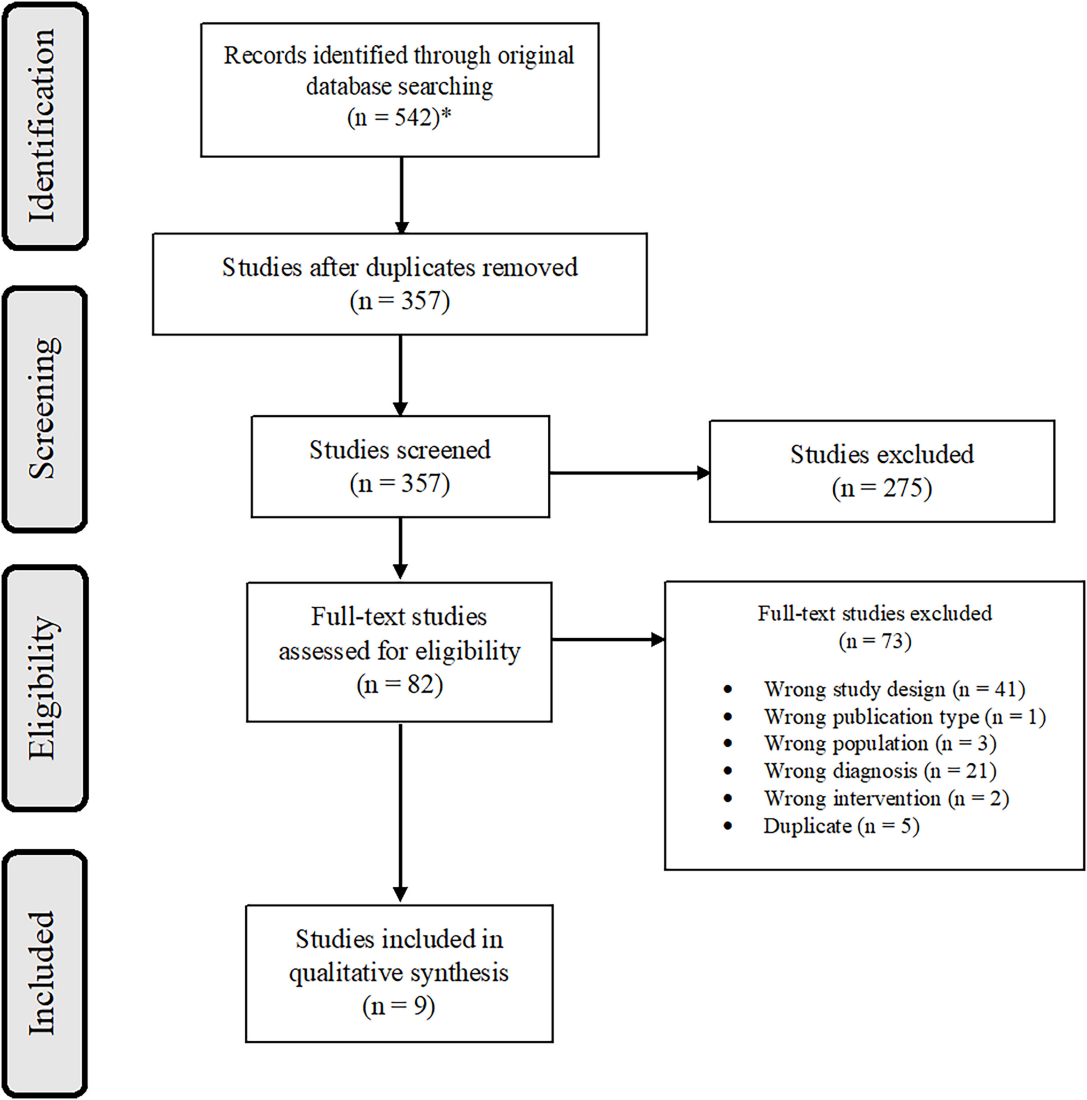
PRISMA flow diagram. *As 538 studies.

Database and hand searches yielded 542 references, reflecting 538 studies. After removing duplicates, the team screened 357 studies. A total of nine studies met eligibility criteria and were subsequently included in the review (Table 2). The PRISMA flow diagram (Figure 1) provides a detailed accounting of exclusions. There were seven studies that used EEG-NF (van der Kolk et al., 2016; Noohi et al., 2017; Nicholson et al., 2020; Fruchtman-Steinbok et al., 2021; Leem et al., 2021; Winkeler et al., 2022; Fine et al., 2023) and two studies that used fMRI-NF (Zotev et al., 2018; Zhao et al., 2023). One of the EEG-NF studies used a sham control (Nicholson et al., 2020) and the rest used passive controls (any control that did not incorporate an active sham comparator group). One EEG study used ILF neurofeedback (Winkeler et al., 2022) and two studies used fMRI inspired EEG-NF (Fruchtman-Steinbok et al., 2021; Fine et al., 2023). Both studies using fMRI-NF used a sham (Zotev et al., 2018) or yoked sham (Zhao et al., 2023) control.

To address KQ1 we conducted meta-analyses of studies to assess post-treatment PTSD symptom change comparing EEG-NF treatment to passive control in five studies (van der Kolk et al., 2016; Noohi et al., 2017; Fruchtman-Steinbok et al., 2021; Leem et al., 2021; Fine et al., 2023) and fMRI-NF to active controls (sham/yoked sham control) neurofeedback in two studies (Zotev et al., 2018; Zhao et al., 2023). We report results for a pre-post correlation of 0.5. For sensitivity analyses with other measures and correlation strengths, see Supplementary Table 1.

**FIGURE 2:**
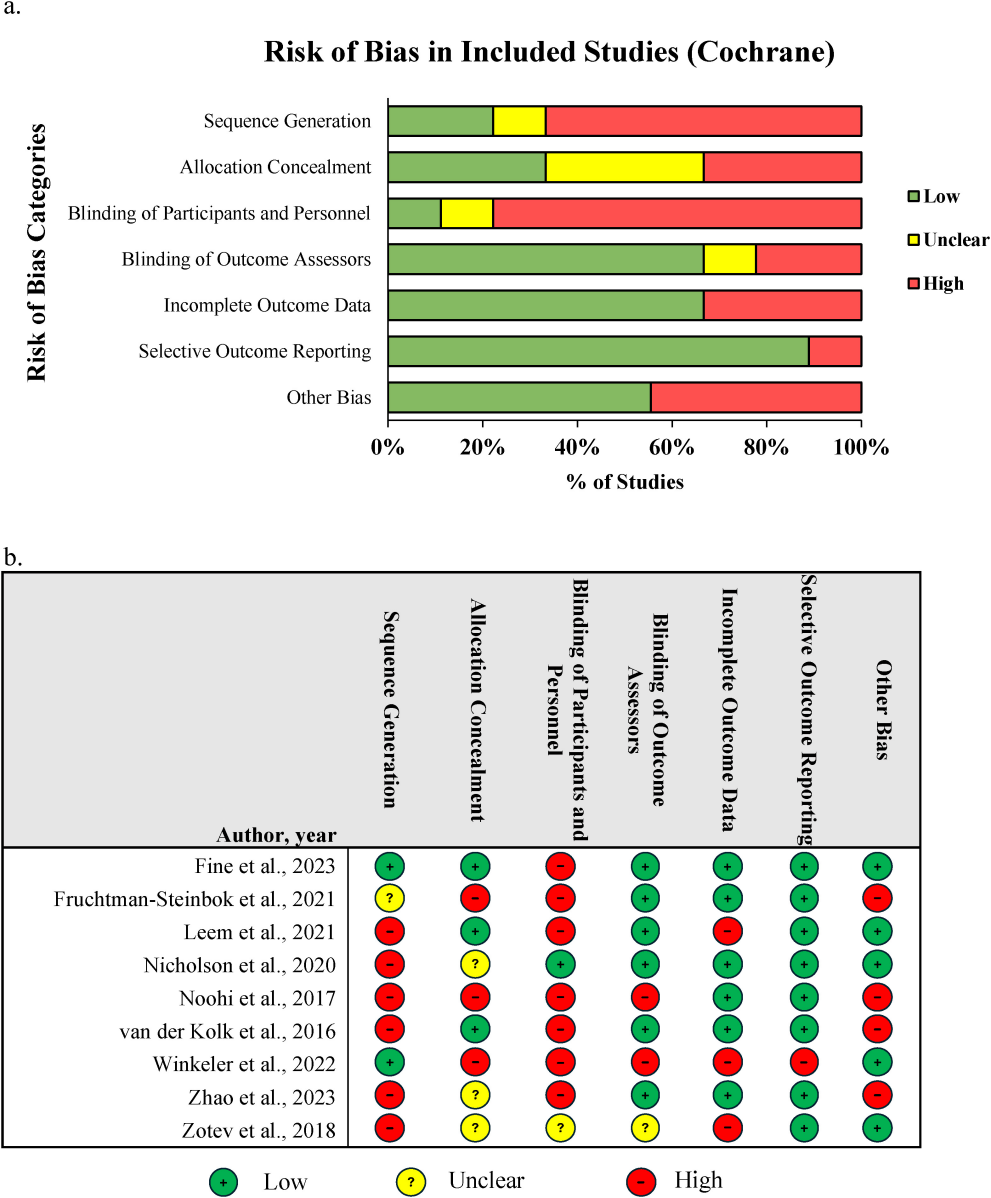
Risk of bias assessments for included studies. **(a)** Summary of risk of bias ratings per Cochrane categories across all studies. **(b)** Individual study risk of bias scores.

Risk of bias ratings varied depending on the source of the bias. The predominant source of high bias across studies was due to limitations with sequence generation and lack of blinding of participants and to a lesser extent the blinding of outcome assessors, allocation concealment, and incomplete outcome data. We also noted that 44% of studies had evidence of other sources of bias, principally related to researchers listed on patent applications for neurofeedback devices and errors in reported data (Figure 2). CRED-nf assessments found that many of the essential items, including online-feature extraction methods, feedback modality and content, hardware and software used, and measures of clinical significance, were consistently reported. Items from the brain outcome measures domain were missing in several studies where assessing clinical efficacy was the primary goal (Noohi et al., 2017; Leem et al., 2021; van der Kolk et al., 2016; Winkeler et al., 2022). Several non-essential, but encouraged items, were only found in the minority of studies or entirely absent, including condition or group effects for artifacts, blinding of outcome assessors and statisticians, reporting the extent in which participants and experimenters remained blinded, and uploading code, raw data, and final values to an open access repository. Results of CRED-nf assessments can be found in the Supplementary Figures 3, 4 and Supplementary Table 2.

### 3.1 EEG-NF versus passive control

**FIGURE 3:**
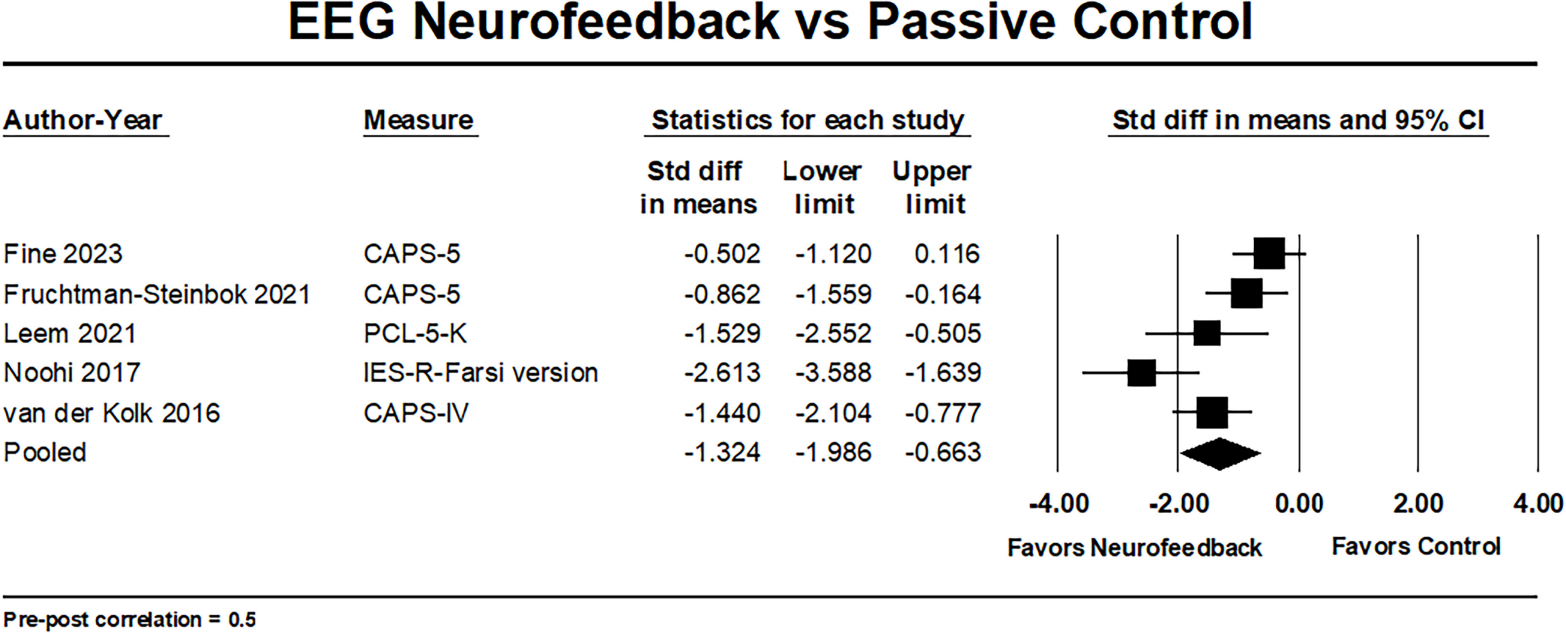
Forest plot of EEG-NF vs. passive controls in pre- to post-treatment PTSD outcomes.

Five studies (van der Kolk et al., 2016; Noohi et al., 2017; Fruchtman-Steinbok et al., 2021; Leem et al., 2021; Fine et al., 2023) compared the effects of EEG-NF to a passive control. Meta-analyses using data obtained from four PTSD outcomes measures (CAPS-DSM-IV, CAPS-5, PCL-5-K [Korean version of the PCL-5], and IES-R-Farsi version) yielded a mean effect size of -1.32 (95% CI: −1.99, −0.66) and a statistical heterogeneity of τ^2^ = 0.41 and I^2^ = 74% (Figure 3). These findings suggest superiority of EEG-NF treatment compared to a passive control condition with a moderate to large effect size. The statistical heterogeneity increased with higher correlation (τ^2^ = 0.26 and I^2^ = 66% when correlation = 0.3 to τ^2^ = 0.65 and I^2^ = 80% when correlation = 0.7). Our confidence in this finding determined using GRADE is very low, meaning that the research does not provide reliable indication of the likely effect, but there is a very high likelihood that the finding will be substantially different. GRADE ratings were downgraded due to concerns over risk of bias (Guyatt et al., 2011e), inconsistency (Guyatt et al., 2011b), imprecision (Guyatt et al., 2011a) and conflicts of interest (Guyatt et al., 2011c; Table 3).

**TABLE 3:** Grading of Recommendations Assessment, Development and Evaluation (GRADE).

Number of studies	Design	Risk of bias	Inconsistency	Indirectness	Imprecision	Other	GRADE* of evidence for outcome
**Comparison: EEG-NF vs. passive control (any non-sham, e.g., WLC, TAU, no treatment)**							
5	RCT	Serious risk of bias (−1)	Serious inconsistency (−1)	No indirectness	Serious imprecision (−1)	COI and potential pub bias (−1)	Very Low^1^
**Comparison: EEG-NF vs. active control (sham or yoked sham)**							
1	RCT	None	N/A	No indirectness	Serious imprecision (−1)	None	Low**
**Comparison: fMRI-NF vs. active control (sham or yoked sham)**							
2	RCT	Serious risk of bias (−1)	No inconsistency	No indirectness	Serious imprecision (−1)	COI (−1)	Very low

COI, conflict of interest; EEG-NF, electroencephalogram neurofeedback; fMRI-NF, functional magnetic resonance imaging neurofeedback; RCT, randomized controlled trial; TAU, treatment as usual; WLC, waitlist control. High = This research provides a very good indication of the likely effect. The likelihood that the effect will be substantially different is low. Moderate = This research provides a good indication of the likely effect. The likelihood that the effect will be substantially different is moderate. Low = This research provides some indication of the likely effect. However, the likelihood that it will be substantially different is high. Very low = This research does not provide a reliable indication of the likely effect. The likelihood that the effect will be substantially different is very high. Substantially different = a large enough difference that it might affect a decision. ^1^Dose response +1. Higher dose improved outcome (see “3.1.1 Meta-regression” section). *GRADE Working Group grades of evidence. **Reduction of one point due to there being one study for this comparison.

#### 3.1.1 Meta-regression

To explore the relationship between treatment duration (dose response) and effect size in the EEG treatment compared to passive condition studies, meta-regression analysis was conducted. The regression coefficient of −1.12 (95% CI: −1.98, −0.26) and intercept of −0.67 indicates that the mean effect size of longer duration studies is larger (associated with greater PTSD symptom reduction) compared to shorter duration studies. This suggests that treatment duration may be associated with larger effect size. The model explained 79% of the variance between studies with a residual heterogeneity of I^2^ = 35%.

### 3.2 EEG-NF versus active control

One study compared EEG-NF to a yoked sham control group. Specifically, EEG-NF relative to sham neurofeedback produced an effect size of −0.43 (95% CI: −1.09, 0.23). This study suggests a small effect size with minimal benefit of EEG-NF over an active control, however, the confidence intervals are inconclusive for this study. Our confidence in this finding determined using GRADE is low. GRADE ratings were downgraded due to concerns imprecision (Guyatt et al., 2011a) and there being only a single study for this comparison (Owens et al., 2010; Table 3).

### 3.3 fMRI-NF versus active control

**FIGURE 4:**
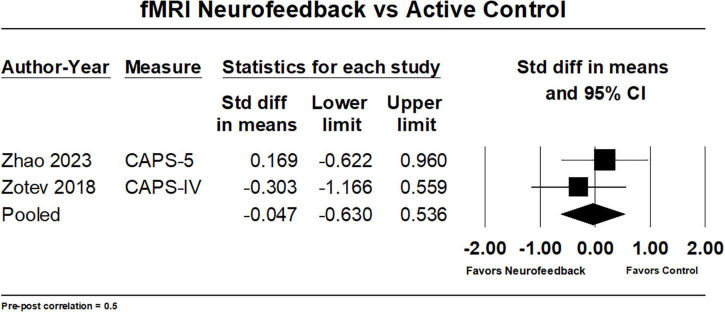
Forest plot of fMRI-NF vs. active controls in pre- to post-treatment PTSD outcomes.

Meta-analysis of two studies comparing fMRI-NF to sham neurofeedback using data obtained from two PTSD measures (CAPS for DSM-IV, CAPS-5) produced a mean effect size of −0.05 (95% CI: −0.63, 0.54) (Figure 4). The results were similar across other values of pre-post correlations. These results do not indicate evidence of benefit of fMRI-NF treatment compared to sham neurofeedback. Due to the small number of studies, we did not have the power to detect heterogeneity. Our confidence in this finding determined using GRADE is very low. GRADE ratings were downgraded due to concerns over risk of bias (Guyatt et al., 2011e), imprecision (Guyatt et al., 2011a), and conflicts of interest (Guyatt et al., 2011c; Table 3).

### 3.4 Any neurofeedback modality versus active control

To analyze the effects of neurofeedback relative to active controls, regardless of the modality of neurofeedback used, we pooled the two fMRI-NF active control studies with the single active control EEG-NF study. We found that neurofeedback relative to active controls produced an effect size of −0.21 (95% CI: −0.65, 0.22) and a statistical heterogeneity of τ^2^ = 0 and I^2^ = 0%. See Supplementary material for the forest plot (Supplementary Figure 1) and sensitivity analysis (Supplementary Table 1). Inclusion of the single EEG-NF study using an active control (Nicholson et al., 2020) alongside the two fMRI-NF studies increased the effect size by 0.16. This suggests a small effect size with minimal benefit of neurofeedback over an active control, however, the confidence intervals are inconclusive.

### 3.5 EEG-NF versus any control

We also pooled all EEG-NF studies (k = 6) and found that EEG-NF relative to any control produced an effect size of −1.16 (95% CI: −1.75, −0.57) and a statistical heterogeneity of I^2^ = 73% and τ^2^ = 0.39. See Supplementary material for the forest plot (Supplementary Figure 2) and sensitivity analysis (Supplementary Table 1). Inclusion of the single EEG-NF study using an active control (Nicholson et al., 2020) alongside the five EEG-NF studies using passive controls reduced the effect size by 0.16. This suggests superiority of EEG-NF compared to any control condition with a moderate effect size.

### 3.6 ILF-NF

One study employed ILF-NF training and consisted of patients with comorbid PTSD and eating disorders (Winkeler et al., 2022). This study could not be included in the meta-analysis due to incomplete reporting of PTSD symptom data. This experiment used the Impact of Event Scale-Revised (IES-R) but only reported means and standard deviations for the hyperarousal and avoidance subscales. No neural measures were reported, and the methodological differences between ILF-NF and the other EEG-NF paradigms precluded a combined analysis. There was a marginal group by time interaction in the reduction of avoidance symptoms observed in the ILF-NF treatment group. No group by time interaction was observed for hyperarousal symptoms.

### 3.7 Neural results

To address KQ2, we synthesized all relevant neural results from the included studies. Four of the 13 studies (including secondary analyses) did not present neural data as part of their results (van der Kolk et al., 2016; Noohi et al., 2017; Leem et al., 2021; Winkeler et al., 2022). All four studies used EEG-NF compared to passive controls and focused on examining clinical changes rather than neural effects of neurofeedback. Table 4 summarizes some key characteristics and findings from studies that reported neural results. Additional information to supplement Table 4 and synthesis of neural outcomes are detailed below.

**TABLE 4:** Key characteristics and neural results of included studies.

References	Neural target	Evidence of target engagement	Pre-post fMRI	Analyses performed	Summary of key findings
Fine et al., 2023	Amygdala by proxy (amyg-EFP, downregulation)	Yes—Improved modulation of amyg-EFP signal as the training progressed	Yes	Brain-clinical correlation	No correlation between amyg-EFP modulation and clinical improvement as measured by CAPS
				Amyg-EFP transfer to amygdala BOLD	Successful transfer of learned downregulation of amyg-EFP signal to amygdala BOLD signal downregulation
				Amygdala BOLD	No between group differences in amygdala downregulation when comparing pre- to post-treatment
Fruchtman-Steinbok et al., 2021	Amygdala by proxy (amyg-EFP, downregulation)	Yes—Improved modulation of amyg-EFP signal as the training progressed	Yes	Brain-clinical correlation	No correlation between amyg-EFP success and clinical improvement as measured by CAPS or PCL
				Amyg-EFP transfer to amygdala BOLD	Successful transfer of learned downregulation of amyg-EFP signal to amygdala BOLD signal downregulation
				Amygdala BOLD and clinical outcomes	Between group differences in amygdala downregulation when comparing pre- to post-treatment; partial correlation analysis of amygdala BOLD downregulation with PTSD symptom change not significant
Nicholson et al., 2020 ^1^	Alpha downregulation	Yes—Alpha rhythm power downregulation improved as training progressed	Yes	Network analyses (connectivity)	Significant intrinsic connectivity network changes of the anterior DMN, posterior DMN, and SN in EG only, no significant group-by-time interactions were found for any ICNs
				Brain-clinical correlation	Larger improvements on CAPS across groups were associated with decreased SMA connectivity with the SN
				Neurofeedback performance-brain correlation	Positive correlation of alpha-down regulation and anterior DMN connectivity with right posterior insula in EG only
Nicholson et al., 2023 ^2^	Alpha downregulation	Yes—see Nicholson et al., 2020	Yes	Conjunction analysis of baseline vs. post-treatment alpha power	Alpha resynchronization in the EG observed in frontal electrodes corresponding to dmPFC
				Pre-post power changes at other EEG bands	EG group: Reduced beta power corresponding to right anterior cingulate and insula Sham group: Reduced delta power corresponding to left precentral gyrus
				Brain-clinical correlation	Absence of group-by-time interaction nor correlation with alpha rebound and pre- to post-treatment CAPS scores
Misaki et al., 2018b ^3^	Left amygdala (upregulation)	Mixed—see Zotev et al., 2018	Yes	rsFC/MDMR	Increased connectivity between the SMA and dACC correlated with symptom change for PCL, but not CAPS, for EG group only; increased connectivity between precuneus and lSFG associated with decreased hyperarousal symptoms of PTSD within EG, no between group effects
Misaki et al., 2019 ^3^	Left amygdala (upregulation)	Mixed—see Zotev et al., 2018	Yes	SEMM whole brain exploratory (brain-clinical effect modeling)	Less activation of the dmPFC and rMCC was associated with greater PTSD symptom improvement. Greater symptom reduction also associated with less activation of precuneus, right SPL and right insula, only for participants that received low neurofeedback signal (regardless of group)
Shaw et al., 2023 ^2^	Alpha downregulation	Yes—see Nicholson et al., 2020	Yes	Network analyses (connectivity) during n-back tasks	Decreased connectivity of the left angular gyrus with the left CEN (1-back) and decreased connectivity of the posterior SN with a cluster overlapping the dlPFC and vlPFC (2-back) for EG in group by session interaction
				Correlation of neurofeedback performance and whole-brain BOLD during 2-back task	Better neurofeedback performance correlated with greater left posterior insula and right TPJ BOLD in EG only
				Correlation of neurofeedback performance and whole-brain BOLD during 1-back task	Better neurofeedback performance correlated with greater dmPFC, right STG, left TPJ, thalamus and cerebellum lobule 6–7 BOLD response in EG only
Zotev et al., 2018 ^1^	Left amygdala (upregulation)	Mixed—Within treatment session showed some upregulation of LA, but EG group declined in ability to upregulate amygdala in final session	No^4^	ROI functional connectivity and brain-clinical correlation	LA-dlPFC connectivity associated with PTSD symptom improvements in EG only
				ROI functional connectivity and brain-clinical correlation	Decreased LA-lingual gyrus connectivity correlated with improved PTSD symptoms in EG only
				Brain-clinical correlation from EEG recordings during fMRI-NF	EG showed increased coherence in upper alpha bands, correlated with both PTSD symptom improvement and LA-dlPFC connectivity in EG only
Zhao et al., 2023	30 most sensitive voxels from localizer of amygdala (downregulation)	Yes—Improved downregulation of amygdala ROI observed post-treatment	Yes	Amygdala BOLD	No change in amygdala downregulation at post-treatment; EG showed improved amygdala downregulation at 30 day follow-up compared to yoked sham control group
				Amygdala rsFC	No significant changes in amygdala connectivity during resting-state at any timepoint
				Brain-clinical correlation	No correlation with PTSD symptom change and amygdala downregulation

^1^Primary study, ^2^secondary study using sample from Nicholson et al., 2020, ^3^secondary study using sample from Zotev et al., 2018, ^4^resting-state fMRI scans were performed at pre-post timepoints relative to neurofeedback training sessions as part of the larger trial, but results were reported separately in Misaki et al., 2018b. Key findings are from Zotev et al., 2018 are from neurofeedback treatment sessions. Amyg-EFP, amygdala electrical fingerprint; BOLD, blood oxygenation level-dependent; CAPS, Clinician-Administered Posttraumatic stress disorder Scale; CEN, central executive network; dACC, dorsal anterior cingulate; dlPFC, dorsolateral prefrontal cortex; dmPFC, dorsomedial prefrontal cortex; EG, experimental group (treatment group); fMRI, functional magnetic resonance imaging; ICN, intrinsic connectivity network; LA, left amygdala; lSFG, left superior frontal gyrus; MDMR, multidimensional distance matrix regression; PCL, Posttraumatic stress Disorder Checklist; rMCC, right midcingulate cortex; ROI, region of interest; rsFC, resting state functional connectivity; SEMM, structural equation model mapping; SMA, supplementary motor area; SN, salience network; SPL, superior parietal lobule, STG, superior temporal gyrus; TPJ, temporoparietal junction; vlPFC, ventrolateral prefrontal cortex.

#### 3.7.1 fMRI-NF

Two fMRI-NF studies, Zhao et al. (2023) and Zotev et al. (2018) examined additional neural correlates of neurofeedback treatment effects. Secondary analyses of the cohort from Zotev et al. (2018), Misaki et al. (2018b), and Misaki et al. (2019) are discussed as well.

Zhao et al. (2023) employed a yoked sham protocol where the neurofeedback signal that participants received followed the same time course as a matched participant in the experimental group. This study found no conclusive changes at either the post intervention or 30-day follow-up in resting-state functional connectivity of the amygdala between the experimental and control groups. It should be noted that the group differences in amygdala activation at the 30-day follow-up were driven by a pattern of increased amygdala activity in the yoked sham control group rather than further downregulation in the experimental group.

Zotev et al. (2018) used the left amygdala (LA) as their target for neurofeedback, while the left horizontal segment of the intraparietal sulcus (LHIPS) was used for the sham-control group. Although there was no conclusive difference between experimental and sham-control group CAPS scores from pre- to post-treatment, the experimental group showed enhanced connectivity between the LA and left dlPFC as treatment progressed, whereas no such effect was seen in the control group. This LA-dlPFC functional connectivity enhancement observed between the first and last neurofeedback session was also negatively correlated with changes in PTSD severity—i.e., PTSD symptoms improved as LA-dlPFC connectivity was enhanced. LA connectivity with the right precuneus, and LA connectivity with a cluster overlapping the right amygdala and parahippocampal gyrus were also found to be negatively correlated with changes in PTSD symptom severity. These effects highlight neural correlates associated with PTSD symptom relief, but not between-group effects of neurofeedback.

Zotev et al. (2018) also employed simultaneous EEG recordings during the fMRI scans. The experimental group showed increased coherence in upper alpha bands, mostly in prefrontal and temporal electrodes. EEG coherence is analogous to functional connectivity in fMRI and measures the synchrony across electrodes (Shaw, 1981; Bowyer, 2016). This increased coherence was correlated with both PTSD symptom improvements and LA-dlPFC connectivity for the experimental group only.

There were several secondary analyses performed on the Zotev et al. (2018) cohort. Misaki et al. (2019) performed whole brain exploratory analyses to see what mediating and/or moderating neural activity is associated with PTSD symptom changes via structural equation model mapping (SEMM). Intriguingly, greater symptom reduction was found to be associated with less activation of precuneus, right superior parietal lobule and right insula, but only for participants that received *low* neurofeedback signal. They found that for these regions, there was a moderating effect of the strength of neurofeedback signal received, for both the sham control and experimental group. Collectively, the findings of Misaki et al. (2019) highlight nonspecific neural effects related to symptom change.

Likewise, Misaki et al. (2018b), used approximately the same cohort as Zotev et al. (2018) along with healthy and veteran control groups from a separate study (Misaki et al., 2018a). Several analyses were performed, including resting-state functional connectivity (rsFC) analyses of areas previously identified to have abnormal rsFC (Misaki et al., 2018a), and a longitudinal multidimensional distance matrix regression (MDMR) analysis. The MDMR analysis, which examined connectivity changes outside of *a priori* seed regions from the rsFC analyses across all PTSD subjects, regardless of treatment condition. The main finding from the MDMR analysis was the observation that increased connectivity between precuneus and left superior frontal gyrus (lSFG) was associated with symptom relief. There were similar trends for both the sham control and experimental groups regarding the precuneus and lSFG when CAPS sub-D scores (hyperarousal symptom cluster) were included in the analyses.

#### 3.7.2 fMRI-informed EEG-NF

Two studies, Fine et al. (2023) and Fruchtman-Steinbok et al. (2021), employed amyg-EFP neurofeedback whereby signals from electrode Pz served as a proxy for amygdala activity. Fine et al. (2023) used a homogenous sample consisting of survivors of childhood sexual abuse with treatment-resistant PTSD undergoing intensive psychotherapy in an inpatient setting. Fruchtman-Steinbok et al. (2021) used a more heterogeneous sample consisting of individuals with PTSD treated in an outpatient setting. Fruchtman-Steinbok et al. (2021) found between-group improvements in CAPS-5 scores pre- to post-treatment for both the neutral and trauma subgroups, but Fine et al. (2023) did not find between-group effects pre- to post-treatment. In both Fine et al. (2023) and Fruchtman-Steinbok et al., 2021, participants were able to transfer the learned downregulation of the amyg-EFP neurofeedback signal to amygdala BOLD signal downregulation in post-treatment scans. While the neural measures of amyg-EFP and amygdala BOLD downregulation were correlated in both studies, Fine et al. (2023) did not find changes in amygdala BOLD signal at the post-treatment phase whereas Fruchtman-Steinbok et al. (2021) did.

#### 3.7.3 EEG-NF

The only primarily EEG-NF study, not informed by fMRI, to present neurophysiological findings was Nicholson et al. (2020), along with secondary analyses done by Nicholson et al. (2023) and Shaw et al. (2023). While alpha down EEG-NF was used for treatment sessions, the primary aim of Nicholson et al. (2020) was to examine ICN changes between their experimental group and yoked sham controls during pre- and post-treatment fMRI scans. Post-treatment changes in the experimental group included decreased precuneus connectivity with the posterior DMN, increased dmPFC connectivity with the anterior DMN and decreased PCC connectivity with the posterior DMN. These connectivity changes suggest a normalization of connectivity away from the previously predominant posterior DMN to anterior DMN after treatment. Larger improvements on CAPS were associated with decreased supplementary motor area (SMA) connectivity with the SN. Despite the normalization of aberrant connectivity in the experimental group, no significant group-by-time interactions were found for any ICNs.

Nicholson et al. (2023) found that the pooled PTSD cohort from Nicholson et al. (2020) exhibited reduced alpha power at baseline compared with healthy controls. This aberrant alpha activity, linked to hyperarousal symptoms of PTSD (Clancy et al., 2017, 2020b), was the neurophysiological correlate of interest. There were noticeable effects on alpha resynchronization, or the “alpha rebound effect” (Kluetsch et al., 2014), specific to the experimental group following treatment. This alpha resynchronization in the experimental group was observed in frontal electrodes, corresponding to dmPFC, a key region of the DMN that plays a role in self-referential processing (Lanius et al., 2015) and emotion regulation (Morawetz et al., 2025). As dmPFC was also implicated in the original analyses (Nicholson et al., 2020), the alpha rebound effect seen here provides additional evidence of restored anterior DMN connections only in the experimental group.

Shaw et al. (2023) examined data from pre- and post-treatment personalized emotional n-back (working memory) tasks collected during the same sessions as Nicholson et al. (2020). Their aims were to explore whole-brain activity and ICN effects during an n-back task. Whole-brain activation during the n-back tasks were also correlated with neurofeedback performance. In addition to results detailed in Table 4, whole-brain analyses found significantly greater activity of the left dlPFC for the experimental group in a group-by-time interaction during all trials of the 1-back portion of the experiment. Shaw et al. (2023) highlight some neural mechanisms in which alpha-down neurofeedback can potentially restore networks while under cognitive load in cases of PTSD, but the association of these ICN changes with PTSD symptom change were not the primary focus of these analyses.

## 4 Discussion

In this systematic review of neurofeedback training for PTSD symptoms, we examined nine RCTs across 13 papers (including secondary studies). Eight studies were meta-analyzed (*N* = 248) in our primary analyses aimed at examining the efficacy of neurofeedback for PTSD compared to active or passive controls using fMRI-NF or EEG-NF (KQ1). We found moderate effects for EEG-NF compared to passive controls, and inconclusive effects for EEG-NF versus active controls and fMRI-NF compared to active controls. Our confidence in EEG-NF compared to passive controls and fMRI-NF compared to active controls was very low. Our confidence in EEG-NF compared to active controls is low. GRADE ratings were primarily downgraded due to concerns over risk of bias, imprecision, and conflicts of interest. EEG-NF and fMRI-NF studies were naturally delineated along passive control and active control study types, respectively, aside from a single active control EEG-NF study. Pooling all EEG-NF studies found moderate effects of neurofeedback and pooling all active control studies found inconclusive effects of neurofeedback. Our synthesis of neural mechanisms (KQ2) found that there could be some nonspecific and network implications of neurofeedback, however, between-group comparisons were rarely conclusive and never associated with PTSD symptom alleviation.

This work builds on previous reviews examining the effectiveness of neurofeedback for PTSD symptomology that presented descriptive results of individual studies without meta-analyses (Chiba et al., 2019; Panisch and Hai, 2020; Pindi et al., 2022); did not link records from the same study together prior to meta-analysis, greatly limiting interpretability (Hong and Park, 2022; Matsuyanagi, 2025); included too few PTSD studies (Ciccarelli et al., 2023); or limited their analyses to EEG-NF (Steingrimsson et al., 2020; Askovic et al., 2023). We have performed a methodologically rigorous examination of potential differences in neurofeedback modality on PTSD outcomes including discussion of the relevant neural outcomes. This systematic review and meta-analysis fills in an important gap in both of these areas. Furthermore, by not pooling studies that utilized active and passive control groups together for our main analysis, our findings illustrate that much of the ostensible effects of neurofeedback training for PTSD reported elsewhere (Askovic et al., 2023; Voigt et al., 2024) are entirely driven by non-placebo-controlled studies. Finally, we provide an impartial analysis of the extant literature, free of conflicts of interest that have been a common underlying source of potential bias in both clinical trials and reviews of this intervention.

### 4.1 Better controls are needed

EEG-NF versus passive controls showed a moderate effect in favor of neurofeedback. However, this result should be tempered considering the context of our other findings. None of the included studies that used an active control group resulted in decreased PTSD symptoms at post-treatment, regardless of modality. The five studies with the largest effect sizes in favor of neurofeedback used a passive control group for the comparator. Previous reviews have noted similar findings as ours across other clinical contexts, where much of the ostensible effects of neurofeedback compared to passive controls are not observed when compared to active controls (Begemann et al., 2016; Thibault and Raz, 2017). The evidence presented here is in line with this sentiment regarding possible placebo effects or indirect mechanisms. This is reinforced by the paucity of between-group neural effects attributable to symptom improvement seen here. We should note that this is a difficult problem to address, because sham and yoked sham control protocols can have drawbacks as well. Incongruency between cognitive states and the feedback received when using a yoked sham can impact motivation and adherence to the treatment task (Sorger et al., 2019). The use of a sham can also be a decrement to a patient’s motivation if they become aware of their lack of volitional control over the feedback signal (Sorger et al., 2019). Alternatively, additional studies comparing neurofeedback to trauma-focused psychotherapy, the current gold-standard in PTSD treatment (Schrader and Ross, 2021), could help elucidate whether the efficacy of neurofeedback for PTSD is on par with established interventions. Nonetheless, the difference in effect sizes between active and passive control study designs warrants the incorporation of more sham controls in future neurofeedback for PTSD training protocols, especially if we want to establish the specific underlying neural mechanisms that neurofeedback acts upon (Micoulaud-Franchi and Fovet, 2018).

### 4.2 Neurofeedback modality effects

EEG-NF and fMRI-NF studies organically differed in the control type used. Aside from one EEG-NF study that used a sham control (Nicholson et al., 2020), all EEG-NF used passive controls, and all fMRI-NF studies used active controls. Additionally, we pooled all EEG-NF studies and found a moderate effect size in favor of neurofeedback, though slightly lower than the passive control EEG-NF studies alone. The effects of this study were more in line with the two other active control studies, both of which utilized fMRI-NF (Supplementary Figure 1). No additional pooling was required to examine the effects of fMRI-NF, as all studies used active controls. Evidence for or against fMRI-NF was inconclusive. It could be that the modality has very little impact on the outcomes relative to the type of control employed, though due to paucity of studies and an absence of any passive control fMRI-NF studies, this observation should be taken with a great deal of caution. The two fMRI-NF studies provided considerably lower doses of neurofeedback in total during the duration of treatment, which could also contribute to the inconclusive findings observed for the fMRI-NF studies. More evidence will be required to infer whether the neurofeedback modality has an effect on PTSD outcomes.

### 4.3 Neural effects

A common theme among the studies included here is that many neural effects are evident only for within-group comparisons of pre- and post-treatment measures for the experimental groups. This is particularly evident in the sham control studies reviewed here. In these studies, the groups receiving genuine neurofeedback do show pre to post connectivity changes that are not observed in their respective sham control comparator groups. This is indeed promising, but between group comparisons failed to find meaningful changes in neural measures in most cases. If there is a meaningful effect, it is likely that larger sample sizes are required to have the power to detect and confidently determine the underlying neural changes associated with neurofeedback for PTSD.

### 4.4 Dosage effects

We found a dose-response relationship for EEG-NF versus passive controls, where longer neurofeedback exposure compared to shorter exposure resulted in greater PTSD symptom alleviation. This evidence supports the efficacy of neurofeedback for treating PTSD but should be tempered due to the low number of studies and our low confidence in the findings. The dose- response relationship observed here can still be influenced by confounding factors (Redelmeier and Zipursky, 2023) but is nonetheless a promising indicator for treatment efficacy. The results of our analysis differed from those previously published (Hong and Park, 2022), likely due to our inclusion of more recent studies and other methodological considerations, particularly the triple counting of trials using the same sample pool. As more studies are conducted, further examination of this potential dose response relationship could potentially help estimate minimum thresholds to see clinical improvement or the presence of a nonlinear relationship of neurofeedback dosage and PTSD symptom improvement.

### 4.5 CRED-nf assessment

The CRED-nf Checklist was developed by Ros et al. (2020) with the aim of improving objectivity and rigor in the design and reporting of neurofeedback studies. Using this tool, we conducted a comprehensive dual-rater CRED-nf assessment of all included RCTs. Five of the nine studies adhered to over 90% of checklist items deemed “essential,” and 67% of studies were pre-registered trials. Items within the domain of “reporting feedback specifications” were generally observed at a high rate across studies, with the least followed item within that domain being the collection and reporting of brain activity variables and/or contrasts displayed to participants (56%). All studies reported online feature extraction (item 4a) and gave some description of the feedback modality and content (item 4c). The four studies that had the lowest overall adherence to CRED-nf were the same four passive control EEG-NF studies that did not report neural results (van der Kolk et al., 2016; Noohi et al., 2017; Leem et al., 2021; Winkeler et al., 2022). Because these studies emphasized clinical outcomes over neural outcomes, brain-related outcome measures (items 5a–c) are absent. Studies conducted prior to the publication of CRED-nf in 2020 were, unsurprisingly, less likely to follow CRED-nf. It is also worth noting that the three studies with the largest effect sizes followed CRED-nf the least and were passive control EEG-NF studies (Leem et al., 2021; Noohi et al., 2017; van der Kolk et al., 2016). Only 44% of studies justified sample sizes or were labeled as feasibility or proof-of-concept studies. Double-blinding was also uncommon (33%), typically due to practical factors that made masking of investigators and/or participants impossible. Further breakdowns of our CRED-nf evaluation for individual studies and general adherence for items aggregated across studies can be found in the Supplementary material. Compared to previously published CRED-nf evaluations of neurofeedback trials for major depressive disorder (Trambaiolli et al., 2021b) and dementia (Trambaiolli et al., 2021a), the studies of PTSD included here were generally more adherent, possibly due to our strict inclusion criteria and increasing *a priori* use of CRED-nf in the study design process. Similar to Trambaiolli et al. (2021b) we found that the fMRI-NF studies followed CRED-nf better than EEG-NF studies, though our findings should be interpreted cautiously given the small number of fMRI-NF studies included here.

### 4.6 Clinical considerations

#### 4.6.1 EEG-NF and fMRI-NF benefits/limitations

EEG-NF offers some clear benefits over fMRI-NF such as costing significantly less and having substantially fewer restrictions for patient accessibility. Individuals with claustrophobia, metal implants, extreme obesity, and movement disorders are excluded from MRI scans. Even without considering the added financial cost, time is a much more limited resource for fMRI as scanning time is typically limited. The downside of EEG with regards to neurofeedback training is the lack of spatial resolution and inability to target specific brain areas without rigorous source localization techniques (Michel and Brunet, 2019). There is unavoidable smearing of the conductive signal due to layers of the scalp and skill, regardless of the density of electrodes (Burle et al., 2015). The implication for neurofeedback is that EEG protocols often lack a true neural target outside of a relatively wide bandwidth of electrical signals. This is particularly problematic for elucidating more mechanistic properties of neurofeedback, hence the use of pre- and post-treatment fMRI scans seen several times in EEG-NF studies included here (Nicholson et al., 2020, 2023; Fruchtman-Steinbok et al., 2021; Fine et al., 2023; Shaw et al., 2023). Recent methodological advances that enable EEG to target specific ROIs by proxy, namely amyg-EFP (Keynan et al., 2016, 2019), have shown a great deal of promise to work around this lack of target specificity with EEG-NF. Amyg-EFP combines the lower cost and accessibility of EEG-NF, relative to fMRI-NF, with improved target specificity, and has shown to be effective reducing PTSD symptoms in a single arm trial (Fruchter et al., 2024) and one study included here (Fruchtman-Steinbok et al., 2021). In the immediate future amyg-EFP is a promising neurofeedback method for treating PTSD in terms of cost and accessibility is, but more RCTs with larger sample sizes and more rigorous controls are required to demonstrate the efficacy of this technique.

#### 4.6.2 Informing treatment

A benefit of neurofeedback as a treatment for psychological disorders is that it can provide very direct neural correlates of the treatment effects in both responders and non-responders. Along those lines, certain individuals simply have higher capacity for neuromodulation than others and are thus, in theory, more likely to benefit from neurofeedback training. A recent study by Gurevitch et al. (2024) used participants from two studies included in this review (Fruchtman-Steinbok et al., 2021; Fine et al., 2023) along with fibromyalgia patients, and healthy controls and found that participants with a high capacity to modulate amyg-EFP signal had improved alexithymia symptoms following 6–15 sessions of neurofeedback (Gurevitch et al., 2024). More evidence is required to establish how neuromodulation capacity interacts with neurofeedback training for PTSD, however. Another recent study found that baseline event related potential features, particularly slow positive wave abnormalities, were predictive of PTSD symptom reduction following neurofeedback treatment (Askovic et al., 2025). In most settings, clinicians and patients alike may never be able to explain why certain treatments worked for some, but not others. However, individualized neural measures such as these can offer insight and guidance into who may respond most successfully to this form of treatment. We suggest further research regarding the interaction of neuromodulatory capacity and treatment outcomes that is more targeted at PTSD symptomology and additional examinations of neural markers predictive of neurofeedback treatment success similar to Askovic et al. (2025), as this can help inform the proper course of treatment at a more individual level.

### 4.7 Limitations

There are some important limitations of this systematic review and meta-analysis to address. To examine KQ1, we intended on having four main groups: EEG-NF versus passive controls, EEG-NF versus active controls, fMRI-NF versus active controls, and fMRI-NF versus passive controls. A complete delineation along modality and active/passive control types was not feasible, since no fMRI-NF RCTs used a passive control and only one EEG-NF RCT used an active control. We cannot be confident to the extent in which modality or comparator type influences outcomes. Our secondary analyses examining all studies with an active control and all studies using EEG-NF attempt to further elucidate this, but the high overlap of fMRI-NF with active control studies and EEG-NF with passive control studies necessitates a cautious interpretation regarding the relative impact of modality and study design. Neural outcomes were only examined in the included RCTs and related analyses. Limiting the neural outcomes to RCTs has the benefit of comparing neural outcomes with controlled comparators. A systematic review of all studies with pre-post comparisons would be more comprehensive and likely uncover additional neural correlates, though these may be less informative from a clinical standpoint. This review also contains a limited number of studies, which therefore also limits the extent of evidence in which conclusions can be based on. More studies, particularly ones that have stronger controls (i.e., sham and yoked sham) are required to strengthen any conclusions on the efficacy of neurofeedback for PTSD. We also limited our main analysis to pre- and post-treatment measures, rather than including follow-up measures, as follow-up measures were very temporally disconnected from treatment and neural outcomes were rarely measured at follow-up. Delayed treatment effects have been observed for neurofeedback training for other disorders (Rance et al., 2018; Goldway et al., 2019) along with neural changes (Sitaram et al., 2017) suggesting that there could be persistent post-treatment remodeling effects following neurofeedback. This is speculative, but worthy of additional examination with respect to PTSD symptom alleviation. Additionally, we only included studies written in English and may have excluded relevant results written in other languages.

### 4.8 Future directions

#### 4.8.1 Amygdala as a target

The amygdala is frequently targeted directly, or by proxy, in neurofeedback for PTSD protocols. Indeed, targeting the amygdala in neurofeedback training for PTSD has a strong theoretical grounding as the amygdala is critical in fear responses (Davis, 1997; LaBar et al., 1998; Whalen et al., 2001; Mendez-Bertolo et al., 2016) and dysregulation of the amygdala is known to be implicated in PTSD (Rauch et al., 2006; Ressler, 2010; Mahan and Ressler, 2012; Iqbal et al., 2023) but there are numerous considerations to keep in mind regarding the functional role of this region. The amygdala plays a critical role in not just fear responses but encoding both positive and negative valence (Janak and Tye, 2015; Beyeler et al., 2018; Pignatelli and Beyeler, 2019). Disruptions to proper valence encoding is thought to be a key feature of PTSD, which can involve biases toward assigning negative valence to otherwise neutral or positive stimuli (Elwood et al., 2007; Bomyea et al., 2016) or attentional biases to negatively valanced stimuli (El Khoury-Malhame et al., 2011; Ashley et al., 2013; Bomyea et al., 2016). This bidirectional valence encoding can complicate neurofeedback protocols that target the amygdala, since therapeutic efficacy is not necessarily determined by increasing or decreasing amygdala activity generally, but rather, modulating amygdala activity appropriately to a given stimulus. In most of the RCTs included in this review that targeted the amygdala directly or by proxy, the goal was to downregulate amygdala activity in response to negative (e.g., traumatic) or neutral stimuli; however, one study (Zotev et al., 2018) used the opposite paradigm, where the goal of the participants was to upregulate amygdala activity in response to a positive stimulus (i.e., happy memories). The implications of this design choice remain unresolved. Other reviews that have synthesized neurofeedback protocols using the amygdala as a target across a broader literature (i.e., emotion regulation) and address these unresolved implications in greater detail (Barreiros et al., 2019; Goldway et al., 2022), but it is still unclear how these design choices can impact the efficacy of neurofeedback for PTSD. This lack of clarity regarding the most effective approach, e.g., “amygdala upregulation to positive stimuli, amygdala downregulation to negative stimuli” and differences between left and right amygdala valence processing (Zotev et al., 2011; Goldway et al., 2022) are fundamental design choices to examine if future studies are going to continue using the amygdala as a target in the treatment of PTSD.

#### 4.8.2 Beyond the amygdala

A large swath of research and RCTs have focused on the amygdala and PFC to amygdala connectivity, but the neural correlates of PTSD extend to numerous brain regions beyond these areas. As pointed out by Misaki et al. (2018b), PTSD symptom reduction was associated more with the normalization of dACC-SMA connectivity than any amygdala connectivity changes. Interestingly, Nicholson et al. (2020) also found that normalization of aberrant SMA connectivity to the larger SN, of which the dACC is considered a central hub (Patel et al., 2012), was associated with PTSD symptom improvement. Furthermore, results from Misaki et al. (2019) suggest that hyperactive dmPFC and rMCC are potential *hindrances* in neurofeedback training success for PTSD. This result is particularly fascinating because it highlights brain areas that diminish treatment effects, rather than areas that improve them, providing potential targets for future neurofeedback training protocols in the treatment of PTSD. The complexity of PTSD and the underlying neural correlates warrants an increased emphasis on regions beyond the amygdala in neurofeedback for PTSD trials.

A notable example of precisely this point is a recent fMRI-NF pilot study that directly compared the efficacy and neural effects for individuals with PTSD when targeting the amygdala vs. PCC for a single training session (Lieberman et al., 2023). This study found that the group trained to downregulate amygdala response when viewing personalized trauma stimuli had no unique neural effects not seen in the group trained to downregulate PCC to the same stimuli. The groups showed equal capacity for downregulating their respective target regions and the PCC group showed improvements in distress symptoms not seen in the amygdala group (Lieberman et al., 2023). This study nicely illustrates the untapped potential for neurofeedback training in PTSD treatment and we recommend more investigations like this are conducted. Moreover, for fMRI-NF, using target areas besides the amygdala can also mitigate commonly cited concerns of poor signal-to-noise ratio due to magnetic inhomogeneity and physiological artifacts arising from the basal vein of Rosenthal (Boubela et al., 2015).

Implementing advanced neurofeedback methodology may also benefit this area of research. Real-time multivoxel pattern analysis (MVPA) neurofeedback, DecNef, has been established and utilized in the treatment of PTSD (Chiba et al., 2019), depression (Yamada et al., 2017) and other disorders and domains (Watanabe et al., 2017). RCTs using DecNef for PTSD are underway (Chiba et al., 2025) and may offer exciting new insights into this treatment modality. Using distributed patterns of neural activity, rather mean activation from a target ROI, has many potential benefits. For example, rather than treating the amygdala as functionally homogenous, researchers and clinicians can employ MVPA to indirectly address the differences in fear, threat and valence processing observed throughout amygdala subregions (Brown et al., 2014). Further, in light of the promising results on the normalization of connectivity networks within the treatment group of Nicholson et al. (2020), connectivity based neurofeedback that targets larger networks rather than a single ROI offers great potential in this field as well (Scheinost et al., 2020). It is also worth mentioning that as 7 Tesla scanners become more common and cost-effective, dynamically updated high precision neurofeedback targets can provide more targeted and individualized neurofeedback treatments (Ganesan et al., 2024). The individualized treatment approaches afforded by these advancements are worth considering for PTSD.

## 5 Conclusion

Neurofeedback in the treatment of PTSD is still an emerging area of investigation and there are some crucial considerations for future research to address. Our certainty of evidence for both EEG-NF versus passive controls and fMRI-NF versus active controls is very low and our certainty of evidence for EEG-NF versus active controls is low. There were only nine included RCTs in this review, several of which had a high risk of bias. Much of this stems from limitations of sequence generation and a lack of adequate blinding of participants and personnel, something very difficult, but still possible, to implement in neurofeedback studies. Higher quality RCTs that utilize more rigorous double-blinding in this domain are required. Only three studies used neurofeedback sham controls (two yoked sham (Nicholson et al., 2020; Zhao et al., 2023), one sham (Zotev et al., 2018)). The rest used passive controls, which are easier to implement and less resource intensive. Much of the evidence in favor of neurofeedback training for PTSD arises from these passive control studies. It is unclear whether neurofeedback modality impacts PTSD outcomes due to heterogeneity between comparator groups employed by fMRI-NF and EEG-NF RCTs. The largest single study included 40 participants in the treatment condition and only three studies (van der Kolk et al., 2016; Fruchtman-Steinbok et al., 2021; Fine et al., 2023) enrolled more than 20 participants into their treatment arms, highlighting the need for much larger sample sizes. Larger studies would improve precision and reduce small-sample bias. Many of the studies included in this review have examined the underlying neural mechanisms associated with the therapeutic effects of neurofeedback training in some form, but several do not report any neural results or data at all (van der Kolk et al., 2016; Noohi et al., 2017; Leem et al., 2021; Winkeler et al., 2022). Treatment groups receiving genuine neurofeedback were frequently observed to have pre- to post-treatment neural changes in connectivity networks and ROI modulation, but these ostensible neural effects were mostly inconclusive relative to comparators. Moreover, neural outcomes were rarely observed to be associated with PTSD symptom relief relative to control groups. Careful considerations need to be made regarding experimental design (e.g., upregulation vs. downregulation of the amygdala, alpha/theta protocol, alpha rhythm protocol, ILF-EEG) and more research needs to be done examining regions besides the amygdala for the purposes of neurofeedback training for PTSD.

## Data Availability

The original contributions presented in this study are included in this article/Supplementary material, further inquiries can be directed to the corresponding author.
