# Supplementary material for: Systematic review and meta-analysis of neurofeedback training efficacy and neural mechanisms in the treatment of posttraumatic stress disorder
Source: Front Neurosci. 2025 Dec 3;19:1658652. doi: 10.3389/fnins.2025.1658652 (PMC12708586; doi:10.3389/fnins.2025.1658652)
Supplement: Supplementary file 1 [file Data_Sheet_1.docx]

**Appendix A**. PubMed search string

(PTSD[tiab] OR “posttrauma*”[tiab] OR “posttrauma*”[tiab] OR “post trauma*”[tiab] OR stress*[tiab] OR “acute stress disorder*”[tiab] OR “combat disorder*”[tiab] OR “war neuros*”[tiab] OR Stress Disorders, Post-Traumatic[MeSH]) **AND** (Neurofeedback OR “EEG biofeedback”[tiab] OR neurotherapy[tiab])

**Appendix B**. Meta-analysis change score formula

$${MeanChange}_{1}= Group 1 mean difference$$

$${MeanChange}_{2}= Group 2 mean difference$$

$$Difference in means = {MeanChange}_{1}- {MeanChange}_{2}$$

$$S{tdMean}_{Diff}= Difference in means / {SD}_{Pooled}$$

**Supplementary Table 1.** Effect sizes and 95% CI with varying pre-post correlation strengths.

| **Treatment** | **Comparison** | **Pre-post correlation** | **Effect Size (95% CI)** | **I^2^ statistic** | **Tau^2^** |
| --- | --- | --- | --- | --- | --- |
| Neurofeedback | Active Control | .3 | -0.19 (-0.62, 0.25) | 0% | 0 |
| Neurofeedback | Active Control | .5 | -0.21 (-0.65, 0.22) | 0% | 0 |
| Neurofeedback | Active Control | .7 | -0.26 (-0.71, 0.18) | 4% | 0.01 |
| fMRI Neurofeedback | Active Control | .3 | -0.04 (-0.62, 0.54) |  |  |
| fMRI Neurofeedback | Active Control | .5 | -0.05 (-0.63, 0.54) |  |  |
| fMRI Neurofeedback | Active control | .7 | -0.06 (-0.65, 0.52) |  |  |
| EEG Neurofeedback | Control | .3 | -0.99 (-1.50, -0.47) | 66% | 0.26 |
| EEG Neurofeedback | Control | .5 | -1.16 (-1.75, -0.57) | 73% | 0.39 |
| EEG Neurofeedback | Control | .7 | -1.48 (-2.21, -0.75) | 81% | 0.65 |
| EEG Neurofeedback | Passive Control | .3 | -1.12 (-1.69, -0.55) | 66% | 0.27 |
| EEG Neurofeedback | Passive Control | .5 | -1.32 (-1.99, -0.66) | 73% | 0.41 |
| EEG Neurofeedback | Passive Control | .7 | -1.69 (-2.51, -0.87) | 80% | 0.68 |

**Supplementary figures**

**Supplementary Figure 1.** Forest plot of standardized pre- to post-treatment mean differences in PTSD outcome measures for any modality of neurofeedback relative to active controls.

**Supplementary Figure 2.** Forest plot of standardized pre- to post-treatment mean differences in PTSD outcome measures for EEG-NF relative to any control group.

**Evaluation of studies on the Consensus on the reporting and experimental design of clinical and cognitive-behavioral neurofeedback studies (CRED-nf checklist)**


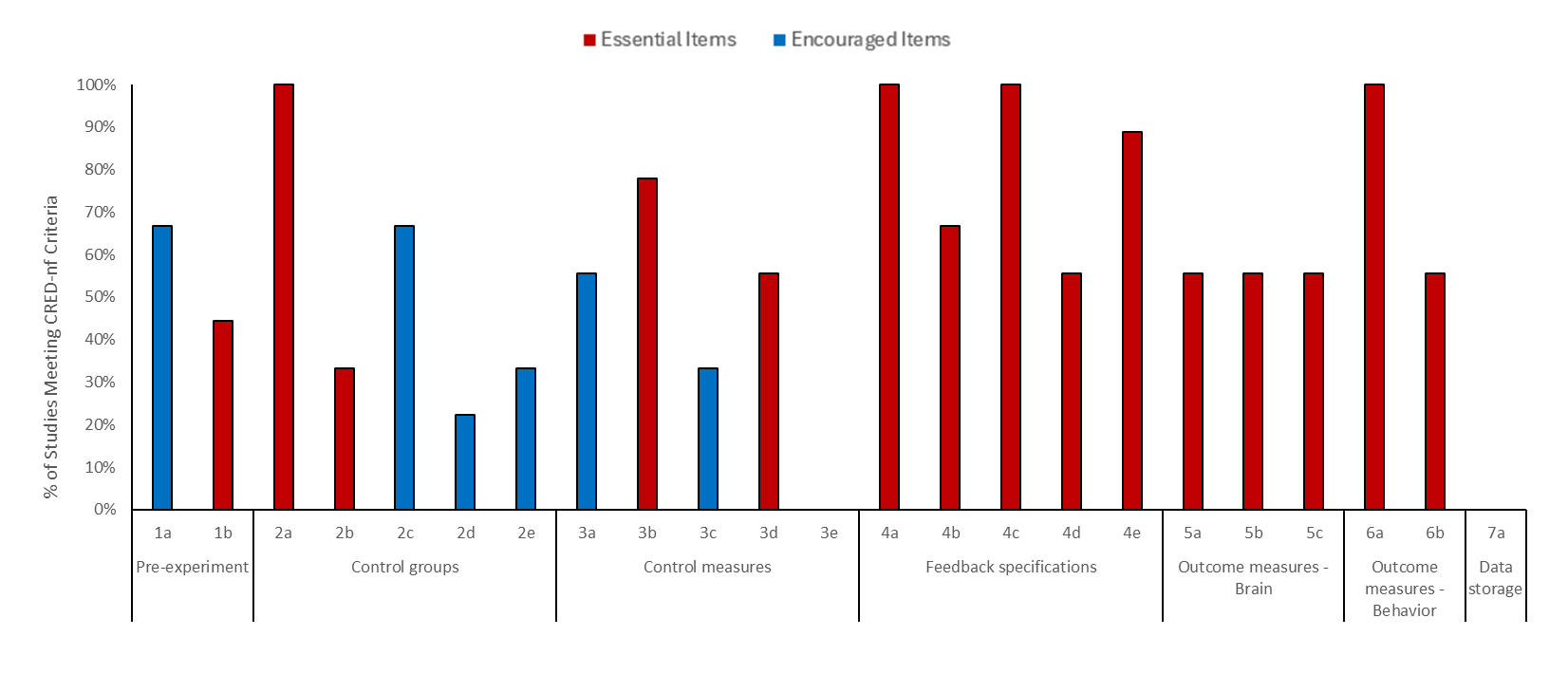


**Supplementary Figure 3.** CRED-nf evaluation pooled across studies

**Supplementary Figure 3 x-axis key:**

1a: Pre-register experimental protocol and planned analyses

1b: Justify sample size

2a: Employ control group(s) or control condition(s)

2b: When leveraging experimental designs where a double-blind is possible, use a double-blind design

2c: Blind those who rate the outcomes, and when possible, the statisticians involved

2d: Examine to what extent participants and experimenters remain blinded

2e: In clinical efficacy studies, employ a standard-of-care intervention group as a benchmark for improvement

3a: Collect data on psychosocial factors

3b: Report whether participants were provided with a strategy

3c: Report the strategies participants used

3d: Report methods used for online-data processing and artifact correction

3e: Report condition and group effects for artifacts

4a: Report how the online-feature extraction was defined

4b: Report and justify the reinforcement schedule

4c: Report the feedback modality and content

4d: Collect and report all brain activity variable(s) and/or contrasts used for feedback, as displayed to experimental participants

4e: Report the hardware and software used

5a: Report neurofeedback regulation success based on the feedback signal

5b: Plot within-session and between-session regulation blocks of feedback variable(s), as well as pre-to-post resting baselines or contrasts

5c: Statistically compare the experimental condition/group to the control condition(s)/group(s) (not only each group to baseline measures)

6a: Include measures of clinical or behavioral significance, defined a priori, and describe whether they were reached

6b: Run correlational analyses between regulation success and behavioral outcomes

7a: Upload all materials, scripts, code, and raw data, as well as final values, to an open access data repository, when feasible


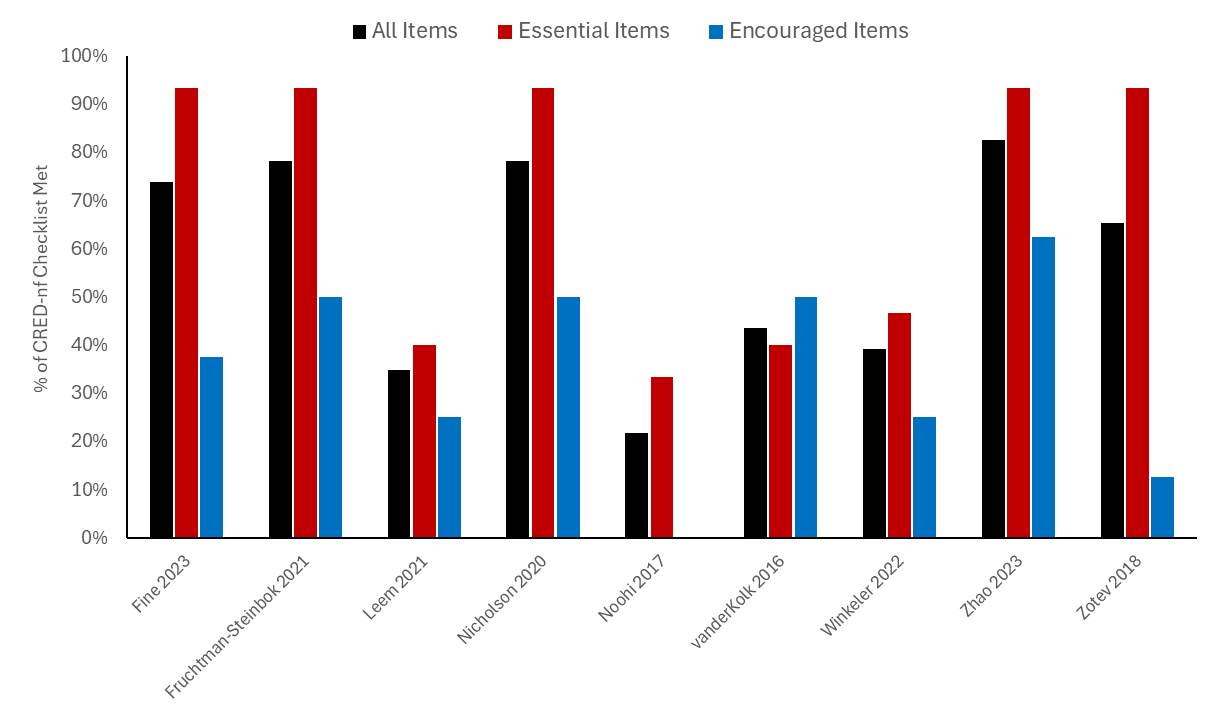


**Supplementary Figure 4.** Individual study results of CRED-nf checklist evaluation

**Supplementary Table 2:** Raw CRED-nf evaluation

- Essential -

- Encouraged -
